# Relationship of established risk factors with breast cancer subtypes

**DOI:** 10.1002/cam4.4158

**Published:** 2021-08-31

**Authors:** Anne Marie McCarthy, Tara Friebel‐Klingner, Sarah Ehsan, Wei He, Michaela Welch, Jinbo Chen, Despina Kontos, Susan M. Domchek, Emily F. Conant, Alan Semine, Kevin Hughes, Aditya Bardia, Constance Lehman, Katrina Armstrong

**Affiliations:** ^1^ University of Pennsylvania School of Medicine Philadelphia Pennsylvania USA; ^2^ Massachusetts General Hospital Boston Massachusetts USA; ^3^ Draper Laboratory Boston Massachusetts USA; ^4^ Newton Wellesley Hospital Newton Massachusetts USA; ^5^ Harvard Medical School Boston Massachusetts USA

**Keywords:** breast cancer, cancer epidemiology, etiology, risk factors, tumor subtypes

## Abstract

**Background:**

Breast cancer is a heterogeneous disease, divided into subtypes based on the expression of estrogen receptor (ER), progesterone receptor (PR), and human epidermal growth factor receptor 2 (HER2). Subtypes have different biology and prognosis, with accumulating evidence of different risk factors. The purpose of this study was to compare breast cancer risk factors across tumor subtypes in a large, diverse mammography population.

**Methods:**

Women aged 40–84 without a history of breast cancer with a screening mammogram at three United States health systems from 2006 to 2015 were included. Risk factor questionnaires were completed at mammogram visit, supplemented by electronic health records. Invasive tumor subtype was defined by immunohistochemistry as ER/PR+HER2−, ER/PR+HER2+, ER, and PR−HER2+, or triple‐negative breast cancer (TNBC). Cox proportional hazards models were run for each subtype. Associations of race, reproductive history, prior breast problems, family history, breast density, and body mass index (BMI) were assessed. The association of tumor subtypes with screen detection and interval cancer was assessed using logistic regression among invasive cases.

**Results:**

The study population included 198,278 women with a median of 6.5 years of follow‐up (IQR 4.2–9.0 years). There were 4002 invasive cancers, including 3077 (77%) ER/PR+HER2−, 300 (8%) TNBC, 342 (9%) ER/PR+HER2+, and 126 (3%) ER/PR−HER2+ subtype. In multivariate models, Black women had 2.7 times higher risk of TNBC than white women (HR = 2.67, 95% CI 1.99–3.58). Breast density was associated with increased risk of all subtypes. BMI was more strongly associated with ER/PR+HER2− and HER2+ subtypes among postmenopausal women than premenopausal women. Breast density was more strongly associated with ER/PR+HER2− and TNBC among premenopausal than postmenopausal women. TNBC was more likely to be interval cancer than other subtypes.

**Conclusions:**

These results have implications for risk assessment and understanding of the etiology of breast cancer subtypes. More research is needed to determine what factors explain the higher risk of TNBC for Black women.

## Graphical abstract

Breast cancer is a heterogeneous disease, with subtypes that may have different etiologies. This study evaluated traditional breast cancer risk factors across tumor subtypes, finding distinct sets of risk factors for each subtype. We additionally discuss implications for screening and risk assessment.

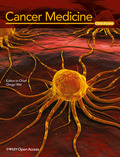

## 1 INTRODUCTION

Breast cancer subtypes are typically classified based on immunohistochemistry according to the expression of estrogen receptor (ER), progesterone receptor (PR), and human epidermal growth factor receptor 2 (HER2). Tumors that are ER and/or PR positive and HER2 negative (ER/PR+HER2−) are the most common, accounting for approximately 73% of breast cancers.^1^ Treatments targeting ER, PR, and HER2 pathways have improved breast cancer outcomes. However, drastic survival differences still exist by tumor subtype, with 5‐year survival near 95% ER/PR+HER2− tumors but just over 75% for triple‐negative breast cancers (TNBC), for which limited targeted therapies exist.^2^, ^3^ Better understanding of the etiologies of each subtype could identify pathways that could be targeted by treatment and preventive interventions.

The existing literature suggests that breast cancer subtypes have unique etiologies.^4^ The canonical breast cancer risk factors used in risk prediction models, such as family history, breast biopsy, and hyperplasia, and reproductive risk factors largely reflect the risk of ER/PR+HER2− breast cancer. Prior studies have produced some inconsistent results with respect to the associations of body mass index (BMI) with the risk of breast cancer subtypes.^4^ Furthermore, studies have differed in their assessment of interactions of BMI and breast density and the interactions of these factors with menopause status, which may explain some of the inconsistency across studies.

The purpose of the study was to compare breast cancer risk factors across breast cancer subtypes, with a specific focus on assessing associations of BMI and breast density and the interactions of these factors with menopause status.

## 2 METHODS

### 2.1 Study population

**FIGURE 1:**
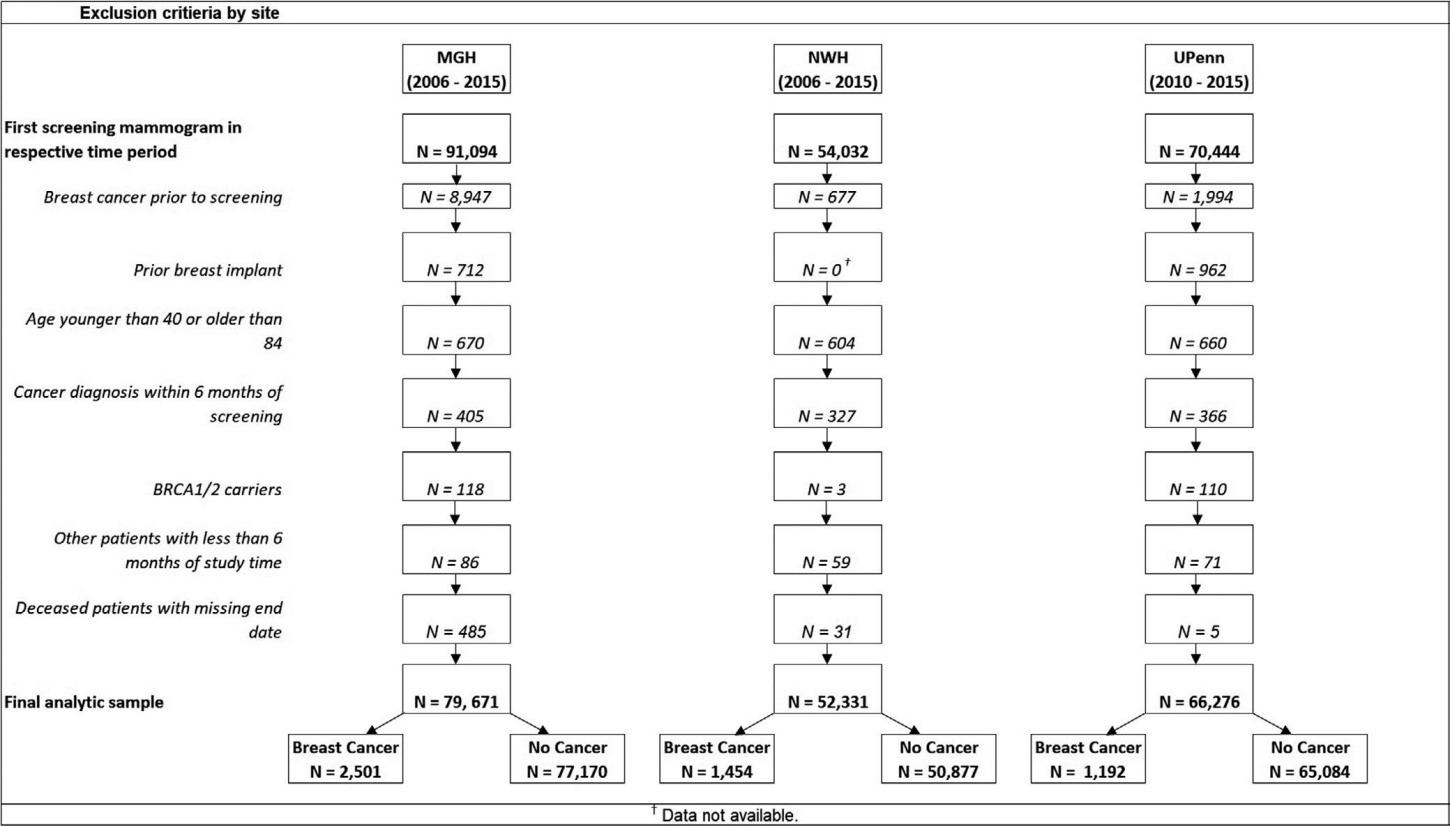
Exclusion criteria by site. Figure shows exclusion criteria for mammography patients across three sites, Massachusetts General Hospital (MGH), Newton‐Wellesley Hospital (NWH), and the University of Pennsylvania Health System (Penn). For all sites, participants with prior breast cancer, prior breast implant, age outside of the 40–84 range, cancer diagnosis within 6 months of screening, BRCA1/2 mutation, patients with <6 months of study time, and deceased patients with missing date of death were excluded. This resulted in a total of 79,671 for MGH, 52,331 patients for NWH, and 66,276 patients for Penn

The study population included women aged 40–84 who received a screening mammogram at Massachusetts General Hospital (MGH) from 2006 to 2015, Newton‐Wellesley Hospital (NWH) from 2006 to 2015, or the University of Pennsylvania Health System (Penn) from 2010 to 2015. Women were excluded from the analysis if they had prior breast cancer, breast implants, or a known *BRCA1*/*2* mutation. We additionally excluded women with less than 6 months of follow‐up, including women diagnosed with breast cancer within 6 months of mammography to maintain temporality between risk factor ascertainment and cancer diagnosis. Finally, women who died but did not have a known date of death or date of the last contact were excluded from analyses. Details of the study populations and exclusions are provided in Figure 1. This study was approved by the University of Pennsylvania Institutional Review Board.

### 2.2 Risk factors

We used a tiered strategy for the assessment of risk factors with the primary source being a risk factor questionnaire completed by the patient at the time of their mammogram. All risk factors were self‐reported, with the exception of age and breast density. Missing information was then supplemented by electronic health records (EHR) when appropriate. If self‐report BMI information was missing, EHR weight and height and/or BMI were used if the body measurement occurred within 1 year prior or within 6 months after screening mammogram (*N* = 3160, 1.6%). Imputation was used to estimate height and then calculate BMI for an additional 18,163 patients (9.2%) that did not have self‐reported or EHR height data but did have weight data, using the median value for that site. Implausible BMI values—those <12 or >82—were considered missing.^5^ Missing information on prior atypical hyperplasia was also extracted from EHR (*N* = 136). Missing information on BI‐RADS (Breast Imaging Reporting and Data System) breast density was extracted from radiology reports using natural language processing as described previously (*N* = 8855).^6^ Since BI‐RADS density category titles changed during the course of the study time, the BI‐RADS 4th edition^7^ density categories (1, 2, 3 or 4) have been translated to the BI‐RADS 5th edition corresponding categories (A, B, C, and D).^8^ Missing prior biopsy information was obtained from linkage to pathology records (*N* = 92). Menopause status was defined based on age and self‐reported menstruation status; patients were automatically considered postmenopausal if they had stopped menstruating or were over 55 years of age, and premenopausal otherwise.^9^

### 2.3 Cancer outcomes

Breast cancer diagnoses through December 31, 2018 were identified from health system cancer registries as well as the Massachusetts, Pennsylvania, New Jersey, and Delaware state cancer registries. Invasive cancers were characterized based on the expression of ER, PR, and HER2 from immunohistochemistry as reported to the cancer registries. In addition, HER2 expression was manually abstracted from EHR for cases diagnosed prior to 2010. Tumor subtypes were defined as ER and/or PR+HER2−, ER and/or PR+HER2+, ER and PR−HER2+, or ER and PR and HER2− (TNBC). Additionally, we categorized whether invasive breast cancer cases were screen‐detected or not screen‐detected for cases diagnosed through 2016. We defined cases as screen‐detected if there was a positive mammogram (BI‐RADS 0, 3, 4, 5) within 1 year prior to cancer diagnosis date. We further defined cases as interval cancers if there was a negative mammogram (BI‐RADS 1, 2) within 1 year of cancer diagnosis, consistent with established definitions.^10^ Cancers that did not have a mammogram within 1 year prior to diagnosis were not coded as screen‐detected or interval (*N* = 902, 37% of invasive cancers).

### 2.4 Statistical analysis

Cox proportional hazards modeling was used to estimate the hazard ratios (HRs) for breast cancer subtypes for each risk factor. We ran separate models with each tumor subtype as the outcome, using a time origin of 6 months post mammogram date, with censoring upon the diagnosis of DCIS (ductal carcinoma in‐situ), other tumor subtypes, death, date last contact for patients with the missing date of death or December 31, 2018 for patients not known to have died. We tested the interactions of BMI with menopause status, breast density with menopause status, and BMI with breast density for each tumor subtype, based on interactions reported in prior studies.^11^, ^12^ Additionally, we also tested the interaction of breast density with race/ethnicity. When testing interactions, breast density was grouped into two categories: non‐dense for those with a density of BI‐RADS A or B, and dense for those with a density of BI‐RADS C or D. Additionally, we examined associations of the number of births with tumor subtypes among the subgroup of parous women. In addition, we performed site stratified Cox models, but since results were similar, un‐stratified models are presented. Missing data were considered to be an additional category in modeling, but estimates are not reported here. Sensitivity analyses were performed after multiple imputations using chained equations (MICE) to evaluate the effect of missing data on our results.^13^ Finally, we performed logistic regression among cancer cases to look at the odds of the cancer being screen‐detected or not, and the odds of the cancer being interval cancer or not according to breast cancer subtypes, adjusted for age, race, atypical hyperplasia, family history, breast density, BMI, and menopause status, factors that have been previously associated with interval cancer risk.^5^, ^14^, ^15^, ^16^ An alpha level of 0.05 was considered statistically significant.

## 3 RESULTS

Table 1 displays the characteristics of the study population. Together, the study population included 198,278 women with a median of 6.5 years of follow‐up (IQR 4.2–9.0 years). Participants had a mean age of 54.3 years at the time of screening. About 11% of participants had ever had a breast biopsy, and 0.9% had previously had atypical hyperplasia. Half of the patients (50.4%) were age 12 or 13 at menarche. The majority of the study population were under the age of 30 at their first live birth, with 5.9% being over the age of 30 at first birth and 21.3% being nulliparous. Most women had 3 or fewer live births, with 8.1% having had 4 or more live births. Most participants (87.3%) had no known family history of breast cancer. The study population was 73.7% white and 15% Black/African American, with the remainder of participants identifying as Hispanic/Latina, Asian/Pacific Islander, or another race. About 38.3% of participants had a BMI under 25 (considered underweight to normal), 24.9% from 25 to 29.9 (overweight), and 25% had a BMI over 30 (considered obese). Postmenopausal women made up a majority of the study population (58.2%). A majority of patients (82.5%) had a BI‐RADS breast density of B or C (described as scattered fibroglandular tissue and heterogeneously dense, respectively), with 7.7% in the BI‐RADS A category (almost entirely fat) and 7.9% in the BI‐RADS D category (extremely dense). There were 4002 invasive cancers diagnosed in the study population, of which 3077 (77.6%) were ER/PR+HER2−, 300 (7.5%) were TNBC, 342 (8.5%) were ER/PR+HER2+, 126 (3.2%) were ER/PR−HER2+, and 157 (3.9%) had missing subtype. In addition, 1042 DCIS cases were diagnosed, which were censored. Of the entire study population, 40.2% came from MGH, 26.4% from NWH, and 33.4% from Penn. The distribution of some risk factors differed across the three subpopulations, as shown in Table S1.

**TABLE 1:** Characteristics of mammography cohort

	All patients *N* = 198,278
	*N* (%) or Mean (SD)
Age at screening (years)	54.30 (11.04)
Age at screening (categories)	
40–49	80,416 (40.6%)
50–59	57,717 (29.1%)
60–69	38,641 (19.5%)
70+	21,504 (10.8%)
Prior biopsies	
0	176,549 (89.0%)
1	17,168 (8.7%)
2 or more	4561 (2.3%)
Atypical hyperplasia	1734 (0.9%)
Age at menarche	
<12	31,802 (16.0%)
12–13	99,978 (50.4%)
14+	47,558 (24.0%)
Missing	18,940 (9.6%)
Age at first live birth	
No births	42,217 (21.3%)
Under 20	20,123 (10.1%)
20–24	36,625 (18.5%)
25–29	41,524 (20.9%)
30 or older	46,077 (23.2%)
Missing	11,712 (5.9%)
Number of relatives with breast cancer	
0	173,075 (87.3%)
1	22,969 (11.6%)
2+	2234 (1.1%)
Race/ethnicity	
White	146,159 (73.7%)
Black/African American	29,822 (15.0%)
Hispanic/Latino	6585 (3.3%)
Asian/Pacific Islander	9371 (4.7%)
Other/unknown	6341 (3.2%)
BMI	
<25	76,035 (38.3%)
25–29.9	49,461 (24.9%)
30+	49,484 (25.0%)
Missing	23,298 (11.8%)
Postmenopausal	115,405 (58.2%)
Number of live births	
0	42,217 (21.3%)
1	41,269 (20.8%)
2	52,116 (26.3%)
3	28,054 (14.1%)
4+	16,032 (8.1%)
Missing	18,590 (9.4%)
BI‐RADS breast density	
BI‐RADS A	15,348 (7.7%)
BI‐RADS B	76,196 (38.4%)
BI‐RADS C	87,459 (44.1%)
BI‐RADS D	15,740 (7.9%)
Unknown	3535 (1.8%)
Site	
MGH	79,671 (40.2%)
NWH	52,331 (26.4%)
Penn	66,276 (33.4%)
Breast cancers	
DCIS	1158 (22.4%)
Total invasive	4002 (77.6%)
Invasive ER/PR+HER2−	3077 (76.9%)
Invasive triple negative	300 (7.5%)
Invasive ER/PR+HER2+	342 (8.5%)
Invasive ER/PR−HER2+	126 (3.2%)
Invasive missing	157 (3.9%)

Abbreviations: ER/PR, estrogen receptor/progesterone receptor; HER2, human epidermal growth factor receptor 2; MGH, Massachusetts General Hospital; NWH, Newton‐Wellesley Hospital.

The associations of known breast cancer risk factors with breast cancer subtypes were assessed using multivariable models (Table 2). For ER/PR+HER2− cancers, all risk factors associations were consistent with the literature and statistically significant. For TNBC, only older age, race, BMI, and breast density were associated with increased risk, with Black women having 2.7 times higher risk of TNBC than white women (HR for age = 1.02, 95% CI 1.02–1.03; race HR for Black women = 2.67, 95% CI 1.99–3.58; HR for overweight women = 1.08–1.96; HR for BI‐RADS category D = 3.37, 95% CI 1.45–7.83). For ER/PR+HER2+ breast cancer, prior biopsy (HR = 1.49, 95% CI 1.08–2.05), atypical hyperplasia (HR = 2.56 95% CI 1.25–4.85), and obesity (HR = 1.59, 95% CI 1.18–2.13) were associated with increased risk. For ER/PR−HER2+, only family history significantly increased breast cancer risk (HR= 1.98, 95% CI 1.29–3.04). Atypical hyperplasia appeared more strongly associated with both HER2+ subtypes than ER/PR+HER2− breast cancer (ER/PR+HER2+ HR = 2.56, 95% CI 1.25–4.85; ER/PR−HER2+ HR = 3.01, 95% CI 0.83–1.99). Higher BI‐RADS breast density was strongly associated with increased risk of all four subtypes, with the largest HRs for ER/PR−HER2+ breast cancer (HR for BI‐RADS D = 6.90, 95% CI 1.35–87.7), though the confidence intervals are wide given the small number of cases in this subtype. Associations of risk factors with combined HER2+ cancers are shown in Table S2. In addition, models estimated using multiple imputations yielded similar results and are displayed in Table S5.

**TABLE 2:** Risk factors for breast cancer subtypes among 198,278 women undergoing screening mammography

	ER/PR+HER2− *N* = 3077			Triple negative *N* = 300			ER/PR+HER2+ *N* = 342			ER/PR−HER2+ *N* = 126		
	HR	95% CI	*p*‐value	HR	95% CI	*p*‐value	HR	95% CI	*p*‐value	HR	95% CI	*p*‐value
Age	1.03*	1.03–1.04	<0.001	1.02*	1.02–1.03	<0.001	1.00	0.99–1.01	0.62	1.00	0.99–1.01	0.689
Race/ethnicity (ref. White)												
Black	0.74*	0.64–0.84	<0.001	2.67*	1.99–3.58	<0.001	0.74	0.50–1.10	0.137	1.28	0.72–2.27	0.400
Other	0.64*	0.56–0.75	<0.001	0.65	0.39–1.07	0.092	0.74	0.50–1.10	0.136	0.82	0.44–1.54	0.541
Prior biopsy (ref. none)												
1+	1.39*	1.26–1.56	<0.001	1.06	0.74–1.52	0.756	1.49*	1.08–2.05	0.014	0.76	0.39–1.46	0.409
Atypical hyperplasia (ref. none)												
Yes	1.47*	1.12–1.92	0.005	0.36	0.05–2.64	0.315	2.56*	1.25–4.85	0.004	3.01	0.83–1.99	0.095
Age at menarche (ref. <12)												
12–13 year	0.93	0.84–1.02	0.112	0.89	0.66–1.21	0.464	0.96	0.72–1.29	0.787	0.72	0.45–1.14	0.163
14+	0.82*	0.73–0.92	0.001	0.76	0.53–1.10	0.143	0.86	0.61–1.21	0.389	0.62	0.36–1.07	0.086
Age first birth (ref. no births)												
<20	0.75*	0.64–0.88	<0.001	1.07	0.71–1.61	0.762	1.16	0.77–1.76	0.469	0.67	0.26–1.68	0.391
20–24	0.87*	0.78–0.97	0.015	0.98	0.69–1.40	0.913	0.70	0.49–1.01	0.057	1.66	0.94–2.92	0.800
25–29	0.92	0.82–1.02	0.108	0.95	0.67–1.34	0.758	0.97	0.71–1.32	0.843	1.41	0.82–2.46	0.211
30+	1.05	0.94–1.15	0.413	0.94	0.49–1.81	0.848	0.97	0.72–1.32	0.849	1.40	0.82–2.39	0.220
Family history^a^ (ref. no family history)												
Yes	1.47*	1.34–1.61	<0.001	1.25	0.91–1.72	0.173	1.32	0.99–1.76	0.063	1.98*	1.29–3.04	0.002
BMI (ref. <25 kg/m^2^)												
25–29	1.32*	1.20–1.45	<0.001	1.46*	1.08–1.96	0.013	1.17	0.88–1.54	0.281	0.89	0.56–1.43	0.638
30+	1.58*	1.43–1.75	<0.001	1.29	0.92–1.80	0.140	1.59*	1.18–2.13	0.002	1.37	0.85–2.22	0.192
Breast density (ref. BI‐RADS A)												
BI‐RADS B	1.56*	1.30–1.87	<0.001	2.76*	1.40–5.47	0.003	1.25	0.72–2.17	0.419	3.77	0.88–47.6	0.069
BI‐RADS C	2.31*	1.93–2.77	<0.001	4.21*	2.11–8.41	<0.001	2.27*	1.30–2.91	0.004	6.73*	1.57–84.9	0.009
BI‐RADS D	2.76*	2.22–3.44	<0.001	3.37*	1.45–7.83	0.005	2.21*	1.15–4.24	0.017	6.90*	1.35–87.7	0.014

Abbreviations: BMI, body mass index; ER/PR, estrogen receptor/progesterone receptor; HER2, human epidermal growth factor receptor 2.

^a^ First degree relatives with breast cancer.

* *p* < 0.05.

As expected based on the previous literature,^11^ there was a significant interaction of menopause status with BMI (*p* < 0.001). Overweight and obesity were more strongly associated with ER/PR+HER2− breast cancer among postmenopausal women than premenopausal women (Table 3; postmenopausal HR for BMI over 30 kg/m^2^ = 1.69, 95% CI 1.50–1.91). Interactions were not statistically significant for the ER/PR+HER2+ or ER/PR−HER2+ breast cancer. Associations of BMI with TNBC were of similar magnitude as seen in other subtypes but were only statistically significant for postmenopausal overweight women (HR = 1.49, 95% CI 1.02–2.01). We also observed a significant interaction between menopause status and breast density for both ER/PR+HER2− (*p* < 0.001) and TNBC (*p* = 0.019), with a stronger association among premenopausal than postmenopausal women (TNBC premenopausal HR for dense breasts = 2.84, 95% CI 1.61–5.04). There was no significant interaction between menopause status and dense breasts for combined HER2+ subtypes (Table S3). There were no statistically significant interactions between BMI and breast density or between race/ethnicity and breast density for any breast cancer subtypes (data not shown).

**TABLE 3:** Interactions of menopause status with BMI and breast density^a^

	ER/PR+HER2−				TNBC				ER/PR+HER2+				ER/PR−HER2+			
	HR	95% CI	*p*‐value	*p*‐interaction	HR	95% CI	*p*‐value	*p*‐interaction	HR	95% CI	*p*‐value	*p*‐interaction	HR	95% CI	*p*‐value	*p*‐interaction
BMI interaction																
Premenopausal	*N* = 872 cases			<0.001	*N* = 86 cases			0.795	*N* = 132 cases			0.101	*N* = 49 cases			0.403
25–29 versus <25	1.22*	1.04–1.44	0.013		1.49	0.90–2.47	0.124		1.41	0.95–2.11	0.091		0.82	0.41–1.66	0.573	
≥30 versus <25	1.22*	1.01–1.48	0.044		1.42	0.78–2.60	0.253		1.31	0.80–2.14	0.283		0.82	0.35–1.90	0.646	
Postmenopausal	*N* = 1830 cases				*N* = 178 cases				*N* = 178 cases				*N* = 69 cases			
25–29 versus <25	1.36*	1.21–1.53	<0.001		1.49*	1.03–2.01	0.036		1.04	0.71–1.53	0.838		0.97	0.52–1.79	0.910	
≥30 versus <25	1.69*	1.50–1.91	<0.001		1.20	0.80–1.81	0.383		1.85*	1.27–2.69	0.001		1.64	0.89–3.01	0.113	
Density interaction^b^																
Premenopausal	*N* = 942 cases			<0.001	*N* = 96 cases			0.019	*N* = 143 cases			0.907	*N* = 50 cases			0.723
Dense versus non‐dense	2.11*	1.76–2.53	<0.001		2.84*	1.61–5.04	<0.001		1.62*	1.04–2.50	0.031		1.91	0.87–4.22	0.109	
Postmenopausal	*N* = 1848 cases				*N* = 196 cases				*N* = 197 cases				*N* = 75 cases			
Dense versus non‐dense	1.47*	1.34–1.62	<0.001		1.38*	1.00–1.03	0.042		1.98*	1.45–2.71	<0.001		2.14*	1.29–3.58	0.003	

Abbreviations: BMI, body mass index; ER/PR, estrogen receptor/progesterone receptor; HER2, human epidermal growth factor receptor 2; TNBC, triple‐negative breast cancer.

^a^ All models additionally adjusted for age, race, prior biopsy, atypical hyperplasia, age at menarche, age a first birth, and family history. Patients missing data on BMI or breast density were excluded from models assessing these interactions.

^b^ Non‐dense breasts comprise BI‐RADS A and B density categories. Dense breasts comprise the BI‐RADS C and D density categories.

* *p* < 0.05.

Among parous women, a greater number of births was associated with reduced risk of ER/PR+HER2− breast cancer (Table 4; HR = 0.95, 95% CI 0.92–0.99). There was no significant association of the number of births with TNBC, ER/PR−HER2+ or all HER2+ cancers (Table S4). There was no association of the number of births as a continuous variable with ER/PR+HER2+ cancers; however, women with two births had a significantly higher risk than women with one birth (HR = 1.39, 95% CI 1.02–1.90), but patients with three or more births had no significant difference in risk than patients with 1 birth (HR = 0.92, 95% CI 0.64–1.32).

**TABLE 4:** Association of number of births with breast cancer subtypes among parous women^a^

	ER/PR+HER2− *N* = 2176			Triple negative *N* = 218			ER/PR+HER2+ *N* = 238			ER/PR−HER2+ *N* = 94		
	HR	95% CI	*p*	HR	95% CI	*p*	HR	95% CI	*p*	HR	95% CI	*p*
Number of births—continuous	0.95*	0.92–0.99	0.011	0.92	0.82–1.03	0.136	1.00	0.89–1.12	0.989	0.91	0.74–1.11	0.341
Number of births—categories (ref. 1)												
2	0.86	0.78–0.96	0.006	0.95	0.68–1.31	0.738	1.39*	1.02–1.90	0.039	0.83	0.51–1.36	0.464
≥3	0.86	0.77–0.96	0.007	0.82	0.58–1.16	0.257	0.92	0.64–1.32	0.640	0.90	0.53–1.52	0.682

Abbreviations: BMI, body mass index; ER/PR, estrogen receptor/progesterone receptor; HER2, human epidermal growth factor receptor 2.

^a^ Additionally adjusted for age, race, prior biopsy, atypical hyperplasia, age at menarche, age first live birth, family history, BMI, and breast density.

* *p* < 0.05.

Table 5 displays the associations of cancer subtypes with screen detection and interval cancers. TNBCs were 33% less likely to be screen‐detected (OR = 0.67 95% CI 0.50–0.88) and more than two times more likely to be interval cancers than ER/PR+HER2− cancers (OR = 2.26 95% CI 1.60–3.20).

**TABLE 5:** Odds of screen detection by breast cancer subtypes among 3744 breast cancer cases diagnosed 2006–2016^a^

	Screen‐detected^b^	Not screen‐detected	OR	95% CI	*p*‐value
ER/PR+HER2−	1274 (56.0%)	1002 (44.0%)	Ref.		
Triple negative	107 (46.1%)	125 (53.8%)	0.67*	0.50–0.88	0.004
ER/PR+HER2+	131 (50.6%)	128 (49.4%)	0.86	0.66–1.18	0.260
ER/PR−HER2+	46 (51.7%)	43 (48.3%)	0.94	0.61–1.44	0.771

	Interval^c^	Not interval			
ER/PR+HER2−	292 (12.8%)	1984 (87.2%)	Ref.		
Triple negative	53 (22.8%)	179 (77.2%)	2.26*	1.60–3.20	<0.001
ER/PR+HER2+	35 (13.5%)	224 (86.5%)	0.94	0.64–1.38	0.743
ER/PR−HER2+	16 (18.0%)	73 (82.0%)	1.26	0.71–2.21	0.432

Abbreviations: ER/PR, estrogen receptor/progesterone receptor; HER2, human epidermal growth factor receptor 2.

^a^ Logistic regression of screen detection adjusted for age, race, atypical hyperplasia, family history, breast density, body mass index, and menopause status.

^b^ Cancers were defined as screen‐detected if there was a positive mammogram within 1 year prior to cancer diagnosis.

^c^ Cancers were defined as interval if they had a negative mammogram within 1 year prior to cancer diagnosis.

* *p* < 0.05.

## 4 CONCLUSIONS

Our results highlight both similarities and differences in risk factors across breast cancer subtypes. Higher breast density was associated with increased risk of all four tumor subtypes, with a stronger association among premenopausal women for ER/PR+HER2− and TNBC. In contrast, the relationship with other risk factors varied across subtypes with distinct sets of risk factors for TNBC (age, race, BMI, and density) and ER/PR+HER2+ (prior biopsy, atypical hyperplasia, BMI, density), ER/PR−HER2+ (family history and density) and ER/PR+HER2− (age, race, prior biopsy, atypical hyperplasia, age at first birth, age at menarche, family history, BMI, and density). Additionally, we found that TNBCs were less likely to be screen‐detected and more likely than other subtypes to be diagnosed as interval cancers. These results have implications both for risk assessment and understanding of the etiology of breast cancer subtypes.

Our results are consistent with a recent large pooled analysis of six cohorts or case–control studies that found that breast density was associated with increased risk of all intrinsic molecular subtypes.^17^ This analysis also observed a significant interaction between percent mammographic density and age for Luminal A cancers, with breast density having a stronger association in younger women. This study observed a similar trend among TNBC that did not reach statistical significance. Furthermore, they found no significant association of breast density with BMI.^17^ Other, smaller studies have yielded inconsistent associations of breast density with breast cancer subtypes.^18^, ^19^, ^20^, ^21^, ^22^, ^23^, ^24^, ^25^, ^26^ Our finding of the interaction of menopause status and BI‐RADS breast density is clinically relevant, as breast density has increasingly been used to identify women who may benefit from supplemental screening, given that mammography is less sensitive among women with dense breasts. There is controversy about the risk‐to‐benefit ratio of supplemental screening for all women with dense breasts, given that nearly half of the screening eligible population has heterogeneously or extremely dense breasts. However, if young women with dense breasts are at particularly high risk for TNBC, which has poor prognosis, supplemental screening may be warranted. Our results are based on a small number of cases among young women, so future studies are needed to validate the large HR that we observed with respect to TNBC in premenopausal women.

While it is well known that Black women have higher risk of TNBC, it is striking that Black women had nearly threefold increased risk even with comprehensive adjustment for breast cancer risk factors in a screened population, a magnitude that has been observed in previous studies which adjusted for fewer risk factors.^27^, ^28^, ^29^ The HR for race was nearly identical prior to multivariable adjustment, suggesting that differences in known risk factors do not explain this disparity. We observed no statistically significant association between age at first birth and risk of TNBC, in contrast to the protective effect for ER/PR+HER2−. This is consistent with three prior studies which also found no significant association of age at first birth with TNBC,^20^, ^30^, ^31^ but contrasts with one prior study which found that older age at first birth was associated with fewer cases of TNBC.^32^ We did not see a significantly increased risk of TNBC among women with greater parity, as has been reported in prior studies.^4^, ^33^, ^34^, ^35^, ^36^ We, unfortunately, lacked data on breastfeeding history in our study, which has shown to be particularly protective against TNBC among women with high parity.^35^, ^36^ As expected based on national data and previous studies,^37^, ^38^, ^39^ Black women had lower risk of ER/PR+HER2− breast cancer compared to White women, as expected based on subtype‐specific incidence rates,^37^ though it is noteworthy that this was true even after adjustment for breast cancer risk factors.

We observed that older age was associated with an increased risk of TNBC. This may seem to be inconsistent with the prior literature reporting younger age to be associated with increased risk of TNBC.^27^, ^39^, ^40^, ^41^, ^42^, ^43^ For example, a large registry‐based study of patients in New Jersey showed that among cancer cases, the OR for TNBC was 1.77 for women aged 20–39, but only 1.10 for women aged 40–49 compared with women aged 50–64.^39^ However, these studies were case only analyses, whereas our study compares women diagnosed with TNBC to women not diagnosed with cancer. While patients with TNBC may be younger than patients diagnosed with other tumor subtypes, TNBC incidence increases with age. Based on SEER estimates, the TNBC incidence rate is 4.0 per 100,000 for women aged 20–39 years compared with 38.9 per 100,000 for women aged 65 and older.^37^ Therefore, our results are not inconsistent with prior data.

We found that prior biopsy and atypical hyperplasia were strongly associated with ER/PR+cancers irrespective of HER2 status but were not associated with TNBC, recognizing that the HR for the association with for ER/PR−HER2+ was relatively large but not statistically significant. Prior biopsy and atypical hyperplasia likely reflect changes in the breast that suggest higher subsequent risk of hormone receptor‐positive and HER2 positive tumors, but these changes do not appear to correlate with TNBC. This finding further points to unique etiologic mechanisms for TNBC.

As expected based on prior studies,^11^ the effect of BMI on ER/PR+HER2− breast cancer differed between premenopausal and postmenopausal women with a greater effect in postmenopausal women. A similar relationship was seen for HER2+ cancers, although the interaction term was not statistically significant (0.07) for ER/PR−HER2+. Most prior studies have found no association between BMI and HER2+ cancers,^32^, ^44^, ^45^, ^46^, ^47^ although one study reported higher risk of HER2+ cancers in overweight women.^42^Although meta‐analyses have found a higher risk of ER− and TNBC among premenopausal obese women,^12^, ^32^, ^45^, ^48^, ^49^, ^50^ BMI was not significantly associated with TNBC in either premenopausal or postmenopausal women in this analysis. A prior analysis of black women found a positive association between obesity and TNBC in premenopausal women but a negative association in postmenopausal women, raising the possibility that the relationship between obesity and menopausal status and TNBC may also vary by race.^51^ Additional studies will be needed to further investigate racial differences in the association of obesity and menopausal status with TNBC.

Our finding that interval cancers are more likely to be triple negative is consistent with existing literature. A population‐based study in Ireland found that triple‐negative tumors were over three times more likely to be interval cancers than screen‐detected.^52^ Similarly, a Canadian population‐based study showed that interval cancers were nearly three times more likely to be ER negative than screen‐detected cancers, though this study lacked data on HER2 status.^53^ One limitation that should be noted is that we lacked information on mammography screening at outside facilities, and therefore our estimates of screen detection and interval cancers may be underestimated. Patients without a mammogram within 1 year prior to their cancer diagnosis were not coded as screen‐detected or interval, which represented 37% of invasive tumors.

The strengths of our study include the prospective design among a large population of women undergoing mammography at three large centers and included the assessment of established breast cancer risk factors along with BMI and breast density, allowing us to assess interactions among risk factors. Additionally, the study includes a significant number of Black women, who are at high risk of dying of cancer but have been underrepresented in research studies to date. The limitations of our study include missing data on some risk factors‐ an inherent problem in studies using data collected for clinical purposes. However, given the prospective design, we do not expect that missing data would be differential by breast cancer diagnosis. We lacked data on the use of hormone replacement therapy (HRT), which is strongly associated with both breast density and breast cancer risk.^54^, ^55^ However, given that the current use of HRT is most strongly associated with risk of ER/PR+HER2− breast cancer and that the prevalence of current HRT use is small,^56^ we do not expect that adjustment for HRT use would greatly affect our results.^20^, ^57^, ^58^ Finally, despite the large study sample, the numbers of TNBC and HER2+ cases were limited.

Our results add to the literature describing differences in risk factors across breast cancer subtypes. We found that breast density may be a particularly strong risk factor for TNBC among premenopausal women, and that the other risk factors evaluated in this study do not explain racial differences in TNBC between Black and white women. These results highlight the urgency of exploring novel risk factors, such as genetics, epigenetics, biomarkers, and environmental exposures to understand the risk for less common but aggressive triple‐negative and ER/PR−HER2+ breast cancer subtypes, as existing risk factors appear largely irrelevant to risk of these tumors.

## CONFLICT OF INTEREST

The authors have the following corporate relationships to disclose: Emily Conant: Dr. Conant has grants and is on the advisory board for iCAD, Inc. and for Hologic, Inc. Kevin Hughes: Dr. Hughes receives honoraria from Hologic (Surgical implant for radiation planning with breast conservation and wire‐free breast biopsy) and Myriad Genetics, Dr. Hughes has financial interests in CRA Health (Formerly Hughes RiskApps) which recently was sold to Volpara. CRA Health develops risk assessment models/software with a particular focus on breast cancer and colorectal cancer. Dr. Hughes is a founder of the company. Dr. Hughes is the Co‐Creator of Ask2Me.Org which is freely available for clinical use and is licensed for commercial use by the Dana Farber Cancer Institute and the MGH. Dr. Hughes's interests in CRA Health and Ask2Me. Org were reviewed and are managed by Massachusetts General Hospital and Partners Health Care in accordance with their conflict of interest policies. Aditya Bardia: Dr. Bardia is a consultant or on the advisory board for Pfizer, Novartis, Genentech, Merck, Radius Health, Immunomedics, Taiho, Sanofi, Daiichi Pharma/Astra Zeneca, Puma, Biotheranostics Inc., Phillips, Eli Lilly, Foundation Medicine. Dr. Bardia is contracted to do research with or has grants (to institution) with Genentech, Novartis, Pfizer, Merck, Sanofi, Radius Health, Immunomedics, Daiichi Pharma/Astra Zeneca. The remaining authors have no conflicts to disclose.

## ETHICAL APPROVAL

This study was deemed exempt from review by the University of Pennsylvania Institutional Review Board.

## Supporting information

Table S1‐S5

## Data Availability

The data underlying this article cannot be shared publicly in order to protect patient privacy. The data may be shared in a de‐identified format on reasonable request to the corresponding author.
