# Supplementary material for: Relationship of established risk factors with breast cancer subtypes
Source: Cancer Med. 2021 Aug 31;10(18):6456–67. doi: 10.1002/cam4.4158 (PMC8446564; doi:10.1002/cam4.4158)
Supplement: Supplementary file 1 — Table S1‐S5 [file CAM4-10-6456-s001.docx]

**Appendix**

| **Appendix Table S1: Characteristics of the study population by site** | | | |
| --- | --- | --- | --- |
|  | **MGH (2006-2015) N=79,671** | **NWH (2006-2015) N=52,331** | **Penn (2010-2015) N=66,276** |
|  | **N (%) or Mean (SD)** | | |
| **Age at Screening (Years)** | 53.8 (11.3) | 52.4 (10.8) | 56.4 (10.6) |
| **Age at Screening (Categories)** |  |  |  |
| 40-49 | 33,836 (42.5%) | 25,429 (48.6%) | 21,151 (31.9%) |
| 50-59 | 22,287 (28.0%) | 14,639 (28.0%) | 20,791 (31.4%) |
| 60-69 | 14,753 (18.5%) | 7,620 (14.6%) | 16,268 (24.5%) |
| 70+ | 8,795 (11.0%) | 4,643 ( 8.9%) | 8,066 (12.2%) |
| **Prior Biopsies** |  |  |  |
| 0 | 78,382 (98.4%) | 43,905 (83.9%) | 54,262 (81.9%) |
| 1 | 854 ( 1.1%) | 6,168 (11.8%) | 10,146 (15.3%) |
| 2 or more | 435 ( 0.5%) | 2,258 ( 4.3%) | 1,868 ( 2.8%) |
| **Atypical Hyperplasia** |  |  |  |
| No | 78,440 (98.5%) | 52,085 (99.5%) | 66,019 (99.6%) |
| Yes | 1,231 ( 1.5%) | 246 ( 0.5%) | 257 ( 0.4%) |
| **Age at Menarche** |  |  |  |
| <12 | 12,715 (16.0%) | 7,973 (15.2%) | 11,114 (16.8%) |
| 12-13 | 38,342 (48.1%) | 29,792 (56.9%) | 31,844 (48.0%) |
| 14+ | 20,492 (25.7%) | 14,243 (27.2%) | 12,823 (19.3%) |
| Missing | 8,122 (10.2%) | 323 ( 0.6%) | 10,495 (15.8%) |
| **Age at First Live Birth** |  |  |  |
| No births | 18,727 (23.5%) | 9,730 (18.6%) | 13,760 (20.8%) |
| Under 20 | 7,405 ( 9.3%) | 2,195 ( 4.2%) | 10,523 (15.9%) |
| 20-24 | 15,705 (19.7%) | 8,321 (15.9%) | 12,599 (19.0%) |
| 20-24 | 15,383 (19.3%) | 13,071 (25.0%) | 13,070 (19.7%) |
| 25-30 | 17,006 (21.3%) | 18,698 (35.7%) | 10,373 (15.7%) |
| 30 or older | 5,445 ( 6.8%) | 316 ( 0.6%) | 5,951 ( 9.0%) |
| **Number of Relatives with Breast Cancer** |  |  |  |
| 0 | 72,071 (90.5%) | 44,657 (85.3%) | 56,347 (85.0%) |
| 1 | 7,096 ( 8.9%) | 7,043 (13.5%) | 8,830 (13.3%) |
| 2+ | 504 ( 0.6%) | 631 ( 1.2%) | 1,099 ( 1.7%) |
| **Race/Ethnicity** |  |  |  |
| White | 65,941 (82.8%) | 46,837 (89.5%) | 33,381 (50.4%) |
| Black/African American | 4,123 ( 5.2%) | 884 ( 1.7%) | 24,815 (37.4%) |
| Hispanic/Latino | 4,190 ( 5.3%) | 550 ( 1.1%) | 1,845 ( 2.8%) |
| Asian/Pacific Islander | 3,963 ( 5.0%) | 2,300 ( 4.4%) | 3,108 ( 4.7%) |
| Other/Unknown | 1,454 ( 1.8%) | 1,760 ( 3.4%) | 3,127 ( 4.7%) |
| **BMI** |  |  |  |
| < 25 | 30,537 (38.3%) | 26,565 (50.8%) | 18,933 (28.6%) |
| 25-29.9 | 19,907 (25.0%) | 13,919 (26.6%) | 15,635 (23.6%) |
| 30+ | 18,067 (22.7%) | 10,792 (20.6%) | 20,625 (31.1%) |
| Missing | 11,160 (14.0%) | 1,055 ( 2.0%) | 11,083 (16.7%) |
| **Menopausal Status** |  |  |  |
| Premenopausal | 33,446 (42.0%) | 25,375 (48.5%) | 24,052 (36.3%) |
| Postmenopausal | 46,225 (58.0%) | 26,956 (51.5%) | 42,224 (63.7%) |
| **Number of Live Births** |  |  |  |
| 0 | 18,727 (23.5%) | 9,730 (18.6%) | 13,760 (20.8%) |
| 1 | 24,466 (30.7%) | 7,729 (14.8%) | 9,074 (13.7%) |
| 2 | 15,657 (19.7%) | 20,193 (38.6%) | 16,266 (24.5%) |
| 3 | 8,570 (10.8%) | 10,383 (19.8%) | 9,101 (13.7%) |
| 4+ | 5,503 ( 6.9%) | 4,296 ( 8.2%) | 6,233 ( 9.4%) |
| Missing | 6,748 ( 8.5%) | 0 ( 0.0%) | 11,842 (17.9%) |
| **BI-RADS Breast Density** |  |  |  |
| Almost entirely fat | 6,130 ( 7.7%) | 949 ( 1.8%) | 8,269 (12.5%) |
| Scattered fibroglandular tissue | 32,770 (41.1%) | 12,331 (23.6%) | 31,095 (46.9%) |
| Heterogeneously dense | 35,367 (44.4%) | 29,152 (55.7%) | 22,940 (34.6%) |
| Extremely Dense | 5,113 ( 6.4%) | 7,266 (13.9%) | 3,361 ( 5.1%) |
| Unknown | 291 ( 0.4%) | 2,633 ( 5.0%) | 611 ( 0.9%) |
| **Breast Cancers** |  |  |  |
| DCIS | 546 (21.7%) | 321 (22.1%) | 291 (24.4%) |
| Total Invasive | 1,955 (78.2%) | 1,133 (77.9%) | 901 (75.6%) |
| *Invasive ER/PR+ HER2-* | *1,552 (79.4%)* | *841 (74.2%)* | *684 (75.9%)* |
| *Invasive Triple Negative* | *136 (7.0%)* | *85 (7.5%)* | *79 (8.8%)* |
| *Invasive ER/PR+ HER2+* | *159 (8.1%)* | *113 (10.0%)* | *70 (7.8%)* |
| *Invasive ER/PR- HER2+* | *63 (3.2%)* | *32 (2.8%)* | *31 (3.4%)* |
| *Invasive missing* | *45 (2.3%)* | *62 (5.5%)* | *37 (4.1%)* |

| **Appendix Table S2: Risk factors for HER2+ breast cancers among 198,278 women undergoing screening mammography** | | | |
| --- | --- | --- | --- |
|  | **All HER2+**  **N=468** | | |
|  | **HR** | **95% CI** | **p** |
| **Age** | 1.00 | 0.99-1.01 | 0.836 |
| **Race/ethnicity (Ref. White)** |  |  |  |
| Black | 0.87 | 0.63-1.20 | 0.402 |
| Other | 0.76 | 0.55-1.07 | 0.113 |
| **Prior Biopsy (Ref. None)** |  |  |  |
| 1+ | 1.27 | 0.96-1.69 | 0.098 |
| **Atypical Hyperplasia (Ref. None)** |  |  |  |
| Yes | 2.65* | 1.50-4.70 | 0.001 |
| **Age at Menarche (Ref. <12)** |  |  |  |
| 12-13y | 0.89 | 0.69-1.14 | 0.343 |
| 14+ | 0.79 | 0.59-1.05 | 0.102 |
| **Age first birth (Ref. No Births)** |  |  |  |
| <20 | 1.05 | 0.72-1,53 | 0.802 |
| 20-24 | 0.90 | 0.66-1.22 | 0.495 |
| 25-29 | 1.07 | 0.81-1.40 | 0.642 |
| 30+ | 1.06 | 0.82-1.38 | 0.652 |
| **Family History^†^ (Ref. No family history)** |  |  |  |
| Yes | 1.48* | 1.17-1.89 | 0.001 |
| **BMI (Ref. 25 kg/m2)** |  |  |  |
| 25-29 | 1.09 | 0.85-1.38 | 0.500 |
| 30+ | 1.52* | 1.18-1.96 | 0.001 |
| **Breast Density (Ref. BI-RADS A)** |  |  |  |
| BI-RADS B | 1.55 | 0.93-2.57 | 0.092 |
| BI-RADS C | 2.78* | 1.67-4.63 | <0.001 |
| BI-RADS D | 2.76* | 1.53-4.98 | 0.001 |
| ^†^ First degree relatives with breast cancer | | | |
| *p < 0.05 | | | |

| **Appendix Table S3: Interactions of Menopause status with BMI and Breast Density for all HER2+ Cancers^†^** | | | | |
| --- | --- | --- | --- | --- |
|  | **All HER2+** | | | |
|  | **HR** | **95% CI** | **p-value** | **p-interaction** |
| **Premenopausal** | *N=181* | | | 0.0595 |
| 25-29 vs. <25 | 1.23 | 0.87-1.74 | 0.249 |  |
| ≥30 vs. <25 | 1.16 | 0.76-1.78 | 0.499 |  |
|  |  |  |  |  |
| **Postmenopausal** | *N=247* | | |  |
| 25-29 vs. <25 | 1.02 | 0.74-1.41 | 0.903 |  |
| ≥30 vs. <25 | 1.79* | 1.30-2.46 | <0.001 |  |
| **Premenopausal** | *N=193* | | | 0.9438 |
| Dense vs. non-dense | 1.68* | 1.15-2.47 | 0.007 |  |
|  |  |  |  |  |
| **Postmenopausal** | *N=272* | | |  |
| Dense vs. non-dense | 2.03* | 1.55-2.65 | <0.001 |  |
| ^†^ Adjusted for age, race, prior biopsy, atypical hyperplasia, age at menarche, age a first birth, and family history. Patients missing data on BMI or breast density were excluded from models assessing these interactions. | | | | |
| *p < 0.05 | | | | |

| **Appendix Table S4: Association of number of births with HER2+ breast cancers among parous women** | | | |
| --- | --- | --- | --- |
|  | **All HER2+ N=332** | | |
|  | **HR** | **95% CI** | **p** |
| Number of births - continuous | 0.97 | 0.88-1.08 | 0.602 |
| Number of births - categories (ref. 1) |  |  |  |
| 2 | 1.20 | 0.92-1.56 | 0.175 |
| ≥3 | 0.91 | 0.67-1.22 | 0.526 |
| *Additionally adjusted for age, race, prior biopsy, atypical hyperplasia, age at menarche, age first live birth, family history, and BMI | | | |
| *p < 0.05 | | | |

| **Appendix Table S5: Risk factors for breast cancer subtypes among 198,278 women undergoing screening mammography, with multiple imputation for missing data** | | | | | | | | | | | | |
| --- | --- | --- | --- | --- | --- | --- | --- | --- | --- | --- | --- | --- |
|  | **ER/PR+HER2-  N=3.077** | | | **Triple Negative  N=300** | | | **ER/PR+HER2+  N=342** | | | **ER/PR-HER2+  N=126** | | |
|  | **HR** | **95% CI** | **p-value** | **HR** | **95% CI** | **p-value** | **HR** | **95% CI** | **p-value** | **HR** | **95% CI** | **p-value** |
| **Age** | 1.03* | 1.03-1.03 | <0.001 | 1.02* | 1.01-1.03 | <0.001 | 1.00 | 0.99-1.01 | 0.686 | 1.00 | 0.98-1.01 | 0.627 |
| **Race/ethnicity (Ref. White)** |  |  |  |  |  |  |  |  |  |  |  |  |
| Black | 0.73* | 0.64-0.84 | <0.001 | 2.63* | 1.96-3.51 | <0.001 | 0.74 | 0.50-1.09 | 0.130 | 1.32 | 0.75-2.33 | 0.400 |
| Other | 0.65* | 0.56-0.75 | <0.001 | 0.65 | 0.40-1.08 | 0.095 | 0.74 | 0.50-1.10 | 0.135 | 0.84 | 0.45-1.58 | 0.586 |
| **Prior Biopsy (Ref. None)** |  |  |  |  |  |  |  |  |  |  |  |  |
| 1+ | 1.40* | 1.26-1.56 | <0.001 | 1.06 | 0.74-1.51 | 0.761 | 1.51* | 1.10-2.08 | 0.011 | 0.76 | 0.40-1.47 | 0.420 |
| **Atypical Hyperplasia (Ref. None)** |  |  |  |  |  |  |  |  |  |  |  |  |
| Yes | 1.47* | 1.12-1.92 | 0.005 | 0.36 | 0.05-2.64 | 0.315 | 2.54* | 1.34-4.81 | 0.004 | 3.04 | 0.83-11.08 | 0.092 |
| **Age at Menarche (Ref. <12)** |  |  |  |  |  |  |  |  |  |  |  |  |
| 12-13y | 0.92 | 0.84-1.02 | 0.103 | 0.89 | 0.65-1.20 | 0.441 | 0.98 | 0.73-1.31 | 0.870 | 0.73 | 0.45-1.18 | 0.198 |
| 14+ | 0.82 | 0.73-0.92 | 0.001 | 0.77 | 0.54-1.11 | 0.156 | 0.87 | 0.62-1.22 | 0.404 | 0.61 | 0.35-1.06 | 0.080 |
| **Age first birth (Ref. No Births)** |  |  |  |  |  |  |  |  |  |  |  |  |
| <20 | 0.75 | 0.64-0.88 | 0.001 | 1.04 | 0.69-1.57 | 0.842 | 1.17 | 0.78-1.77 | 0.449 | 0.66 | 0.26-1.68 | 0.380 |
| 20-24 | 0.89 | 0.79-0.99 | 0.030 | 0.95 | 0.67-1.34 | 0.769 | 0.72 | 0.50-1.03 | 0.069 | 1.60 | 0.90-2.84 | 0.110 |
| 25-29 | 0.93 | 0.83-1.03 | 0.172 | 0.91 | 0.65-1.28 | 0.591 | 0.98 | 0.72-1.34 | 0.843 | 1.33 | 0.77-2.29 | 0.304 |
| 30+ | 1.05 | 0.95-1.17 | 0.320 | 0.85 | 0.59-1.22 | 0.387 | 0.98 | 0.73-1.33 | 0.912 | 1.31 | 0.77-2.23 | 0.322 |
| **Family History^†^ (Ref. No family history)** |  |  |  |  |  |  |  |  |  |  |  |  |
| Yes | 1.47* | 1.34-1.62 | <0.001 | 1.25 | 0.91-1.72 | 0.172 | 1.33 | 1.00-1.79 | 0.052 | 2.01* | 1.31-3.09 | 0.001 |
| **BMI (Ref. < 25 kg/m2)** |  |  |  |  |  |  |  |  |  |  |  |  |
| 25-29 | 1.34* | 1.22-1.48 | <0.001 | 1.50 | 1.11-2.02 | 0.008 | 1.18 | 0.90-1.56 | 0.281 | 0.87 | 0.54-1.41 | 0.575 |
| 30+ | 1.60* | 1.44-1.77 | <0.001 | 1.36 | 0.97-1.92 | 0.078 | 1.60* | 1.17-2.18 | 0.003 | 1.35 | 0.83-2.19 | 0.220 |
| **Breast Density (Ref. BI-RADS A)** |  |  |  |  |  |  |  |  |  |  |  |  |
| BI-RADS B | 1.59* | 1.33-1.91 | <0.001 | 2.78* | 1.40-5.56 | 0.004 | 1.24* | 0.72-2.16 | 0.435 | 3.77 | 0.90-15.71 | 0.069 |
| BI-RADS C | 2.41* | 2.00-2.89 | <0.001 | 4.31* | 2.14-8.67 | <0.001 | 2.29* | 1.31-3.99 | 0.003 | 6.81* | 1.62-28.53 | 0.009 |
| BI-RADS D | 2.94* | 2.35-3.66 | <0.001 | 3.49* | 1.48-8.22 | 0.004 | 2.27* | 1.17-4.40 | 0.016 | 6.90* | 1.47-32.24 | 0.014 |
| ^†^ First degree relatives with breast cancer | | | | | | | | | | | | |
| *p < 0.05 | | | | | | | | | | | | |
